# Targeting CCL2 inhibits viral DNA accumulation and induces APOBEC3A expression in HIV-1 infected primary human macrophages

**DOI:** 10.1186/1742-4690-10-S1-P30

**Published:** 2013-09-19

**Authors:** Michela Sabbatucci, Alessandra Mallano, Cristina Purificato, Daniela Angela Covino, Maurizio Federico, Stefano Vella, Sandra Gessani, Mauro Andreotti, Laura Fantuzzi

**Affiliations:** 1 Department of Hematology, Oncology and Molecular Medicine, Istituto Superiore di Sanitá, Rome, Italy; 2 Department of Drug Research and Medicine Evaluation, Rome, Italy; 3 AIDS National Center, Rome, Italy

## Background

Monocyte/macrophages are reservoirs capable of producing replication-competent HIV virions for many years despite suppressive HAART. APOBEC3 proteins represent critical determinants of monocyte resistance to HIV-1 infection, and their decreased expression during macrophage differentiation results in a permissive target cell population. Chemokines and their receptors are deeply involved in HIV-1 infection control. Monocytes/macrophages are the major source of CCL2 *in vitro* and *in vivo.* Growing evidences suggest that CCL2 plays important roles in AIDS pathogenesis. We previously reported that CCL2 expression is up-regulated during monocyte differentiation to macrophages and is further increased in HIV-1 infected cells, where it acts as an autocrine factor promoting HIV-1 replication. The aim of this study was to investigate the mechanisms by which CCL2 affects HIV-1 replication in macrophages.

## Methods

Macrophages were differentiated from CD14^+^ monocytes isolated from the peripheral blood of healthy donors. Cells were treated with anti-CCL2 or control-antibodies, and infected with HIV-1 _BaL_. Viral infection was assessed by measuring total HIV-1 DNA by qPCR and determining p24-Gag-antigen levels. VSV-G pseudo-typed HIV-1 was used to investigate the effect of CCL2 blocking on viral entry. APOBEC3 family member and SAMHD1 mRNA and protein levels were determined by qPCR and western-blot, respectively.

## Results

We found that CCL2 neutralization induces a strong reduction of HIV-1 DNA level 4 and 7 days post-infection. The effect of anti-CCL2 Ab on viral DNA was concentration-dependent and statistically significant with respect to that of a control Ab. This effect correlated with a strong reduction of the amount of p24 Gag antigen positive macrophages. Since CCL2 blocking also reduced infection with VSV-G pseudo-typed HIV-1, inhibition of HIV-1 infection was not due to a block of viral entry. Treatment of macrophages with anti-CCL2 Ab also resulted in induction of APOBEC3A expression, but did not affected the expression of the other APOBEC3 family members and of SAMHD1. Furthermore, the presence of anti-CCL2 Ab reduced the percentage of p24 Gag Ag positive cells at similar extent both in the absence and in the presence of exogenous dNTPs, thus demonstrating that altered SAMHD1 expression or function cannot account for the CCL2 neutralization-mediated restriction of HIV-1 replication in macrophages.

## Conclusions

Our data demonstrate that targeting CCL2 exerts its antiviral effect at the level of viral DNA accumulation, and that this effect may be linked to induction of APOBEC3A expression. The observation that CCL2 is an endogenous negative regulator of APOBEC3A in macrophages highlights a new mechanism which may contribute to the regulation of the expression of innate intracellular viral antagonists *in vivo.* Thus, this study will potentially lead to identification of new targets for therapeutic intervention to prevent the onset of new reservoirs cells.

